# Leisure as Catalyst for Well-Being and Sense of Belonging Among Iranian Immigrant Women Aged 30–55 in Ottawa, Canada

**DOI:** 10.3390/ijerph23080999

**Published:** 2026-07-30

**Authors:** Mahsa Hadidi, Eileen O’Connor, Nadia Abu-Zahra, Laura Ambrosio, Emily Regan Wills

**Affiliations:** 1 School of Human Kinetics, Faculty of Health Sciences, University of Ottawa, Ottawa, ON K1N 6N5, Canada; 2 School of International Development and Global Studies, Faculty of Social Sciences, University of Ottawa, Ottawa, ON K1N 6N5, Canada; 3 Official Languages and Bilingualism Institute, Faculty of Arts, University of Ottawa, Ottawa, ON K1N 6N5, Canada; 4 School of Political Studies, Faculty of Social Sciences, University of Ottawa, Ottawa, ON K1N 6N5, Canada; emily.wills@uottawa.ca

**Keywords:** immigrant working women, leisure, wellbeing, belonging, social connection, community-based activities, Iranian immigrants, Canada, mental health promotion, settlement

## Abstract

**Highlights:**

**Public health relevance: How does this work relate to a public health issue?**

- Leisure participation is a modifiable social determinant of mental health and well-being for Iranian immigrant women aged 30 to 55 in Ottawa, yet it remains largely absent from settlement programming and public health frameworks targeting newcomer populations.
- Structural constraints including time poverty, transportation difficulties, caregiving demands, weather, and limited culturally accessible information systematically limit participation in this population.

**Public health significance: Why is this work of significance to public health?**

- Community-based leisure activities designed to be low-cost, culturally familiar, and delivered through trusted settlement organizations were associated with immediate benefits that participants described: emotional relief from settlement stressors, social connection, and growing familiarity with the city.
- These outcomes correspond directly to protective factors against acculturative stress, depression, and social isolation, conditions that disproportionately affect newcomer immigrant women.

**Public health implications: What are the key implications or messages for practitioners, policy makers and/or researchers in public health?**

- Settlement organizations, municipal recreation systems, and public health planners should recognize community-based leisure as a legitimate, fundable component of newcomer wellbeing support rather than an optional supplement to settlement services.
- Programming aimed at immigrant working women’s mental health must address structural access conditions, including scheduling, affordability, language, and trusted communication, rather than treating them as incidental obstacles to otherwise adequate provision.

**Abstract:**

Background: Immigrant working women’s well-being is shaped not only by formal health and settlement services but also by everyday opportunities for rest, social connection, cultural expression, and participation in community life. This study examined how three community-based leisure activities were experienced by Farsi-speaking Iranian immigrant women aged 30 to 55 living in Ottawa, Canada. Methods: Organized in partnership with PAND Settlement Services, the activities included a group walk along the Rideau Canal, a Classical Persian dance workshop, and a group visit to the National Gallery of Canada. Data were drawn from 32 post-activity evaluation surveys, researcher field notes, and two follow-up semi-structured feedback sessions. Structured items were analyzed descriptively; open-ended responses and feedback sessions notes were analyzed thematically following Braun and Clarke’s six-phase approach. Results: Participants rated all three activities positively across enjoyment, accessibility, and social contact. Leisure was experienced as a meaningful break from routine, a source of emotional relief, an opportunity to meet other women, and a setting in which shared language and migration experience made participation easier and more comfortable. Sustained participation remained shaped by time pressure, caregiving demands, weather, transportation, cost, and access to clear and trusted information. Conclusions: Leisure activities contributed to the well-being and sense of belonging for the women who participated in the study.

## 1. Introduction

Immigrant settlement is frequently discussed in relation to employment, housing, language acquisition, and access to formal health and social services. While these dimensions matter, they do not fully account for how well-being is constructed and sustained in everyday life. A growing body of scholarship shows that immigrant well-being is also shaped by social inclusion, access to community resources, and the practical ability to participate in meaningful daily activities beyond work and survival-oriented responsibilities [1,2]. For immigrant women in particular, well-being is closely tied to social support, family connectedness, and opportunities to participate in community life; all aspects that can be strained when connections are disrupted through migration [3,4]. Periods of heightened isolation and reduced access to support networks, such as those experienced during the COVID-19 pandemic, have been associated with lower levels of well-being among immigrants, a reminder of how sensitive mental health is to everyday social connections [5].

Leisure is one domain through which broader dimensions of wellbeing become particularly visible. Leisure is increasingly understood as a meaningful social and health-related practice connected to emotional restoration, quality of life, relationship-building, and a fuller sense of self [6,7]. In Canadian research, Suto [8] identified leisure as an important contributor to immigrant women’s well-being, especially where it created space for enjoyment, autonomy, and social connection. More broadly, research on immigrants and leisure demonstrates that recreational and community activities can support adaptation, reduce acculturative stress, strengthen settlement satisfaction, and create opportunities for connection to both people and place [9,10,11,12,13,14]. A recent scoping review further concluded that leisure remains an underutilized component of settlement infrastructure, despite consistent evidence of its contributions to well-being and integration [15].

At the same time, access to leisure is far from equal. Research on women and leisure has long documented how time poverty, family responsibilities, social expectations, and unequal access to resources constrain participation [16,17]. These patterns are often intensified in migration contexts. Studies with immigrant and refugee women identified multiple, overlapping constraints, including limited time, transportation challenges, financial pressure, lack of information, unfamiliarity with local opportunities, cultural expectations, and discomfort in mainstream activity settings [2,18,19,20]. In the Canadian context, newcomers also described recreation and leisure as difficult to access when information is fragmented, programming is not culturally responsive, or participation depends on resources that are not evenly distributed [20,21,22]. Leisure scholarship has long theorized these limits through the concept of leisure constraints. This literature distinguishes between structural constraints such as time, cost, transportation, and the organization of programming; interpersonal constraints, which arise from relationships and social obligations including caregiving and the availability of companions; and intrapersonal constraints such as internalized expectations, confidence, and beliefs about whether leisure is appropriate or deserved [16,17]. Importantly, constraints are not understood as fixed barriers that simply prevent participation. Women negotiate constraints, drawing on facilitators such as flexible scheduling, affordable options in proximity, trusted information, and the support of family or peers so that participation is often adapted, or made partial rather than abandoned altogether [16,17]. Identifying constraints and facilitators provides an important vantage to examine the factors that limited participation but also what made leisure possible for the study participants.

While structural, interpersonal and intrapersonal constraints may affect women’s leisure broadly, they take on distinct forms in the context of migration. Theories on immigrant adaptation describe how newcomers negotiate unfamiliar institutions, languages, and social norms while rebuilding networks and local knowledge that enables participation in social and community life [17,23]. Layered onto the gendered constraints that native-born women also face, immigrant women encounter migration-specific conditions such as limited familiarity with local leisure systems, information that is fragmented or arrives through channels they do not yet use, the loss of established social networks, discomfort in mainstream settings, and the demands of acculturation. These intersect alongside factors such as language, residency status, and racialization that can further restrict access and inclusion [22,23,24,25]. These intersecting conditions mean that opportunities available to native-born women may remain difficult to locate, afford, or feel welcoming for newcomer women, even when formal provision exists. For many immigrant women, leisure is further shaped by gendered norms and family expectations carried across the migration process so that constraints experienced before migration are reconfigured rather than removed in their new settings [21]. Understanding immigrant women’s leisure therefore requires attention to how gender and migration intersect.

Iranian immigrant working women are a population for whom these considerations resonate. Existing work has shown that Iranian women’s leisure is shaped by gendered norms, family expectations, and structural constraints both before and after migration [18]. Research on Iranian immigrants in Canada has highlighted the importance of social determinants, isolation, and community connection in shaping mental health and everyday well-being [24]. Yet there is limited qualitative research examining how structured, community-based leisure activities are experienced by Iranian immigrant working women in a Canadian urban setting, and how such activities may support well-being, connection, and belonging in practice.

This manuscript addresses that gap by drawing on the activity-based component of a broader doctoral study conducted in collaboration with PAND Settlement Services in Ottawa. Three structured leisure activities were organized for Farsi-speaking Iranian immigrant women aged 30 to 55: a group walk along the Rideau Canal, a Classical Persian dance workshop, and a group visit to the National Gallery of Canada. Rather than treating leisure as background context, this study examines how participants described these activities in relation to emotional relief, social connection, familiarity with the city, and the practical conditions that made participation easier or harder. The study was guided by two questions: (1) How did participants experience the three leisure activities in relation to well-being, connection, and belonging? (2) What constraints and facilitators shaped participation across the three activities? Following leisure studies scholarship, we use the term constraints rather than barriers throughout the manuscript. Constraints better reflects that these conditions are not necessarily fixed obstacles that prevent participation altogether; they may ease, intensify, or recede over time, and women often negotiate them to take part fully or partially [17].

## 2. Materials and Methods

### 2.1. Study Design and Community Partnership

This manuscript draws on the activity-based component of a doctoral research project on leisure and community services as potential pathways to well-being among Iranian immigrant working women aged 30–55 in Ottawa, Canada. This project emerged from the research team’s SSHRC-funded community engaged project in partnership with PAND Settlement Services. The activity component of this doctoral study included input from community members at every stage. This partnership-based approach aligns with community-based and participatory health research principles that emphasize trust, responsiveness, and the value of working through established community relationships [23,25], as well as emerging work in leisure studies demonstrating that co-designing programming with community members can strengthen both participation and sense of belonging [26]. In practice, this community input reflected the study’s community-based approach: the choice, timing, format, and location of the activities were guided by community members’ stated preferences, everyday constraints and accessibility needs, gathered through a preliminary leisure interest survey administered through Google Forms (Google LLC, Mountain View, CA, USA) and ongoing consultation with PAND Settlement Services organization [27]. Many of the women who later attended the activities and contributed evaluation data completed the earlier preliminary leisure interest survey, so activities reflected the interests and preferences of the community they were intended to serve.

### 2.2. Participants and Recruitment

Participants were Farsi-speaking Iranian immigrant women aged 30 to 55 living in Ottawa, Ontario, Canada. Recruitment was carried out through bilingual posters circulated via PAND Settlement Services’ communication channels, including newsletter emails and the Telegram messaging platform (Telegram Messenger Inc., Dubai, United Arab Emirates). Separate posters were prepared for each activity and included the date, time, location, eligibility criteria, registration process, and consent requirements. All materials were available in both English and Farsi, and participation was voluntary, based on self-registration. The study focused on this age range because women aged 30 to 55 typically balance paid employment with caregiving and household responsibilities. This period in immigrant women’s lives is often less examined than younger cohorts such as newcomer children, adolescents or students, or studies on older adults. The descriptor working women is used in an inclusive sense throughout the manuscript, referring to women engaged in paid work alongside substantial domestic and caregiving labour, rather than as a formal labour-force screening category.

Across the three activities, 12 post-activity surveys were completed after the group walk, 11 after the dance workshop, and 9 after the gallery visit, for a total of 32 surveys. As participants could choose which activities to attend, the survey totals represent activity-level responses rather than a count of unique individuals. Two women who had attended all three activities later participated in follow-up semi-structured feedback sessions. As the post-activity surveys were anonymous, individual respondents could not be identified across activities, so the extent of overlap in attendance between activities cannot be determined. An overlap that could be identified with certainty was the two women who attended all three activities and took part in the follow-up feedback sessions. For the same reason, women who attended an activity but did not complete a survey could not be tracked, so some degree of self-selection among respondents cannot be ruled out. The 32 surveys therefore represent 32 activity-level evaluations across the three activities rather than repeated measures from a single fixed group. The two follow-up feedback sessions were conducted as individual, one-on-one interviews rather than as focus groups, one with each of the two women who participated in all three activities.

### 2.3. Leisure Activities

Three leisure activities were organized between October and December 2025. The first was a group walk along the Rideau Canal, held on 26 October 2025. The second was a Classical Persian dance workshop, held on 19 November 2025, at a Community Center. The third was a group visit to the National Gallery of Canada during its Free Thursday Nights program on 4 December 2025. All activities were facilitated in Farsi. Each session followed a consistent sequence: participant arrival, informed consent, activity participation, and completion of an anonymous post-activity evaluation survey before departure. Field notes were recorded in Microsoft Word (Microsoft 365, version 2607; Microsoft Corporation, Redmond, WA, USA) by the lead researcher after each session. The walk covered approximately 2.5 to 3 km round trip and lasted about 1.5 h at a relaxed, moderate pace, walking on a well-paved pedestrian pathway with few physical obstacles. Participants stopped at the halfway point for a pause to sit on the benches, drink water, and pose on the Rideau Canal bridge for a group photograph. The weather was mild and sunny, with little to no wind and no rain. The outing concluded with informal tea and conversation.

The three activities differed intentionally in format. The walk provided a low-cost, low-pressure outdoor experience in a familiar public space. The dance workshop created a culturally familiar, movement-based group setting. The gallery visit offered access to a major cultural institution and to free public programming. Together, these formats were designed to explore how different types of leisure might support or constrain participation among immigrant women.

### 2.4. Data Collection

Data were drawn from three complementary sources: post-activity evaluation surveys, field notes, and follow-up semi-structured feedback sessions.

The post-activity evaluation surveys were modelled after team members’ comparable research projects using post leisure activity surveys with Iranian newcomer women over the age of 55. The survey contained both structured and open-ended items. Structured items addressed four areas across all activities: evaluation of the activity and its organization; experiences of well-being and social interaction; perceived constraints on leisure participation; and preferences for future programming. Open-ended items invited participants to share their motivations for attending, valued aspects of the activity, experiences of social connection, growing familiarity with Ottawa, and suggestions for future programs. All surveys were available in English and Farsi and responses submitted in Farsi were translated into English before analysis. To gather data on constraints, questions included asking about conditions that generally make leisure participation more difficult for the respondent, rather than only about the specific activity just completed, and were paired with the item on preferences for future programming to inform subsequent activities. The survey used here was pretested through consultation with the doctoral supervisor. Following that consultation, the instrument was adapted by simplifying and clarifying item wording, adjusting the list of leisure constraints to reflect the circumstances of working women aged 30 to 55, and expanding the open-ended prompts on well-being, social connection, and familiarity with the city.

Field notes were recorded by the lead researcher following each activity. These captured contextual and procedural dimensions not fully reflected in survey responses, including group atmosphere, patterns of interaction among participants, and observations about how the session unfolded in practice.

Two follow-up semi-structured feedback sessions were conducted online through Zoom (version 6.3; Zoom Video Communications, Inc., San Jose, CA, USA), in Farsi language, with women who had attended all three activities, each lasting approximately 45 min. Semi-structured feedback sessions were selected because it offers a flexible framework that keeps the conversation oriented toward the research questions while allowing participants to introduce their own meanings, emphases, and priorities [28]. The follow-up feedback sessions questions were organized thematically, moving from general experiences of leisure and daily routines through participants’ responses to each specific activity and concluding with reflections on constraints, community connection, and programming preferences. Follow-up prompts invited participants to elaborate on specific moments, describe what made participation easier or harder, and reflect on how the activities compared to one another. Participants did not consent to audio-recording. Accordingly, the lead researcher took detailed notes throughout each feedback session. At the close of each session, these notes were reviewed aloud with the participant to verify accuracy and completeness before being translated into English. Although audio-recording might have preserved additional nuance, this approach respected participants’ preferences and ensured that the documented content faithfully reflected what had been shared. Emerging themes were member-checked with the supervisor. Self-reflection to identify potential bias was implemented and addressed, given the lead researcher’s positionality as an Iranian international student in this age cohort.

### 2.5. Data Analysis

Structured survey responses were entered into Microsoft Excel (Microsoft 365, version 2607; Microsoft Corporation, Redmond, WA, USA) and analyzed descriptively using frequencies and percentages. Given the small, activity-specific samples, the quantitative material was used to identify and summarize response patterns rather than to support inferential analysis.

Open-ended survey responses and feedback sessions notes were analyzed thematically following Braun and Clarke’s [29,30] six-phase approach: familiarization with the data, generation of initial codes, construction of candidate themes, review and refinement of themes, definition and naming of themes, and production of the analytical narrative. Analysis began with repeated reading of all translated material, followed by systematic coding across the full dataset and the gradual development of themes capturing recurring meanings and patterns across activities. Field notes were incorporated as supporting interpretive material, particularly when clarifying how the structure, atmosphere, or logistical conditions of a session shaped participants’ experience.

### 2.6. Rigor and Trustworthiness

Several strategies were employed to support the rigor and trustworthiness of the findings. First, data were drawn from three complementary sources, namely surveys, field notes, and feedback sessions, allowing patterns identified in one source to be contextualized and examined against the others. This triangulation strengthened confidence in the themes that emerged across the dataset. Second, member checking was built into the follow-up feedback sessions process: notes taken during each feedback session were reviewed and confirmed by participants before the session closed, reducing the risk of misrepresentation. Third, the use of bilingual materials throughout the study, including recruitment posters, consent forms, surveys, and follow-up feedback sessions facilitation entirely in Farsi, supported meaningful participation and reduced language-related barriers to expression. Finally, the lead researcher maintained reflexive awareness of her position as a member of the Iranian diaspora conducting research within a community, she was also part of, attending carefully to how this dual role might shape data collection, analysis, and interpretation.

### 2.7. Ethical Considerations

Written informed consent was obtained from all participants before participation in each activity and in advance of the follow-up feedback sessions. Consent forms were bilingual and clearly explained the purpose of the study, the voluntary nature of participation, participants’ right to withdraw at any time without consequence, and the steps taken to protect confidentiality. Written informed consent was obtained from all participants involved in the study. Signed consent forms were stored securely and separately from the anonymous post-activity evaluation surveys, which contained no identifying information. Feedback consultation materials were handled confidentially and reported without identifying details. The study was conducted in accordance with the Declaration of Helsinki and approved by the University of Ottawa Research Ethics Board (Protocol H-03-23-8562).

## 3. Results

### 3.1. Activity Overview and Post-Activity Evaluations

Participants positively rated all three activities. For the outdoor walk, all 12 respondents agreed or strongly agreed that they had enjoyed the activity, found registration and participation easy, felt comfortable during the session, and would recommend the route to others. Eleven of the 12 respondents said the walk fit their schedule, and 11 indicated they would be likely to walk the same route or a similar activity again. All 12 respondents agreed or strongly agreed that they felt good or happy during the activity and had made at least one new acquaintance.

The dance workshop received equally strong, positive responses. All 11 respondents agreed or strongly agreed that they enjoyed the session, found it easy to attend, were satisfied with the facilitation, felt encouraged to participate regardless of prior dance experience, and felt good or happy while participating. Ten respondents agreed or strongly agreed that the pace and level of instruction were appropriate, and eight said the workshop increased their awareness of leisure opportunities in the broader community.

The gallery visit was also rated highly. All nine respondents agreed or strongly agreed that they enjoyed the visit, were satisfied with the facilitation, and found the gallery activities engaging. Eight respondents said the visit fit their schedule and that the gallery environment supported their participation. Eight respondents also agreed or strongly agreed that the visit increased their interest in art and culture, encouraged interest in other museums or cultural spaces, and raised their awareness of free-admission programming. Table 1 provides an overview of the three activities. Table 2 presents selected structured survey findings. Table 3 summarizes the most frequently reported constraints across activities.

### 3.2. Leisure as Self-Care and Relief

Across the surveys and follow-up feedback sessions, participants described leisure as something considerably more than simple enjoyment. The activities were consistently associated with relief, lightness, and a temporary release from the demands of daily routine. In the follow-up feedback sessions, leisure was described in plainly affective terms as ‘free time without responsibility’ and ‘time that is not duty.’ These characterizations reveal that leisure was understood not primarily as a type of program, but as a qualitatively different experience of time, one not fully claimed by work, caregiving, household tasks, or continuous obligation.

This understanding appeared across all three activities. Participants used affective language to describe the effects of participation: feeling ‘good,’ ‘happy,’ ‘more active,’ ‘lighter,’ or ‘cheered up.’ One respondent in the dance workshop wrote that the session gave her ‘a lot of positive energy’ after a busy day. Another explained simply that ‘it cheered me up a lot.’ In the gallery survey, one participant described attending because she wanted ‘to do something different and take a break from my repetitive daily routine, work, home, sleep,’ while another wrote that she came because she needed ‘a break from the responsibilities of motherhood and being a wife.’ These accounts suggest that the value of leisure was not only in the activity itself but also for the respite it offered from the demands of daily life. The follow-up feedback sessions reinforced this point. One participant reflected that the activities reminded her that she needed ‘something outside routine, even if small.’ Another, speaking about the gallery visit, remarked that the experience helped her realize she was ‘not just [a] cashier.’ Taken together, these responses show that the activities created brief but meaningful openings in which participants could momentarily step outside heavily scheduled lives and experience a different relationship to time, role, and self.

### 3.3. Activity Format Shaped Leisure Experiences

Although all three activities were experienced positively, participants did not describe them in identical terms. Each format supported a distinct kind of leisure experience.

The outdoor walk was described as especially relaxed, easy, and low-pressure. It required no special preparation, involved no prerequisite skill, and unfolded in a public outdoor environment that felt welcoming and accessible. The side-by-side movement of walking also appeared to facilitate conversation without pressure or social obligation. Survey respondents highlighted ‘walking and chatting with people,’ ‘chatting with new friends,’ and ‘enjoying the beautiful pathway’ as among the most valued aspects of the experience. This made the walk particularly effective for early relationship-building and gentle social participation.

The dance workshop generated a more energized and culturally resonant experience. Participants valued the music, movement, shared cultural references, and the workshop’s supportive atmosphere. One respondent joined because she wanted ‘to learn Persian classic dance,’ while another said the activity helped her feel connected to Iranian culture. Several women emphasized the pleasure of ‘dancing together and talking,’ and others described the session as both physically and emotionally beneficial. One participant wrote that ‘after long time, it made me feel active,’ while another noted it improved her mood ‘by improving blood circulation and boosting mood.’ The workshop also appeared to build confidence: one woman stated, ‘I don’t feel shy anymore,’ and another said she felt ‘more courageous to dance compared to before.’

The gallery visit offered a calmer and more reflective type of leisure. Participants described the setting as peaceful, creative, and inspiring. Open-ended responses emphasized ‘the calm and inspiring atmosphere of the gallery,’ ‘the collection of the art,’ and having ‘a few peaceful hours’ in a quiet environment. The visit also had a distinct place-based dimension: it introduced several women to a major cultural institution in Ottawa and to free-entry programming they had not previously known about. One follow-up feedback sessions participant noted that the experience motivated her ‘creatively.’ These patterns indicate that format mattered in meaningful ways. The walk supported ease and unforced social interaction; the dance workshop created culturally familiar and embodied engagement; and the gallery visit offered calm reflection and cultural exploration. The value of leisure programming lay partly in the diversity of forms through which participants could engage.

### 3.4. Leisure as Social Connection

A central finding across the dataset was that all three activities created structured opportunities for social contact. Participants consistently identified interaction with others as one of the most meaningful aspects of participation. This pattern appeared in both the structured survey items and the open-ended responses: across activities, nearly all respondents agreed or strongly agreed that making new acquaintances mattered to them and that they had made at least one during their session.

The qualitative material adds depth to this pattern. Walk participants highlighted ‘walking and chatting with people,’ ‘chatting with new friends,’ and ‘making friendship’ as important parts of their experience. In the dance workshop, participants described the value of ‘chatting afterward,’ ‘chatting with friends over tea,’ and meeting women who shared similar experiences. One participant wrote that she appreciated ‘hearing our immigration stories,’ while another said the women were ‘very friendly and warm.’ The gallery visit also facilitated new contacts: one respondent explained, ‘As a newcomer I didn’t know anyone, but now I’ve made some new connections that may continue.’

Shared language played a central role in making these interactions easier. One walk participant noted the activity helped her feel connected because ‘we were speaking in our mother tongue,’ while a gallery respondent similarly wrote that ‘speaking in our mother tongue helped.’ In the follow-up feedback sessions, participants suggested that shared language and shared migration experience reduced the social effort required to connect, with one woman stating that connection came naturally ‘because we all speak the same language and have kind of the same experiences.’

At the same time, connection was not uniform. A smaller but important subset of responses indicated that not every activity felt equally welcoming for everyone. In one activity, a participant wrote: To be honest, the group didn’t feel very friendly or warm this time.’ Another said she was unable to make a connection during the visit. The follow-up feedback sessions data complicated the picture further: one participant remarked, ‘If I have a friend, I go; if not, I cancel,’ suggesting that a familiar companion was sometimes a prerequisite for participation. Another noted that she ‘prefers mixed spaces sometimes,’ indicating that an exclusively Iranian group setting was not automatically welcoming for all. These responses do not undermine the broader finding that leisure supported social connection; rather, they show that group-based connection was meaningful in this age cohort but contingent and perhaps shaped by individual personality, prior relationships, and the particular social dynamics of each activity format.

### 3.5. Familiarity with Ottawa and Emerging Belonging

Beyond enjoyable social moments, the activities helped participants become more familiar with Ottawa and, in some cases, more at ease within the city. This process appeared in both direct and indirect ways. Some respondents noted that the activities introduced them to places they had not previously visited, while others described how participation expanded their awareness of community resources and made the city feel more accessible.

The walk along the Rideau Canal provided a striking example. For several women, it was their first time on that route, and they described the outing as a way of coming to know Ottawa more intimately. One respondent wrote, ‘it was my first time walking along the Rideau Canal and was more rewarding with the beauty of the fall season.’ Another said that ‘visiting [a] new place of the city made me feel part of the city.’ The gallery visit had a similar effect. Multiple respondents noted they had not previously visited the National Gallery because of time, cost, transportation, or the absence of someone to go with. Learning about free-admission evenings shifted the gallery from something distant or costly into something imaginable and reachable. The walk ended with tea and conversation which gave participants the opportunity to socialize after the outing.

Participants connected these leisure activities to a broader sense of belonging. In the follow-up feedback sessions, one woman explained that experiences like these ‘teach how [the] systems work and introduce community spaces beyond work and school.’ This observation captures an important dimension of the findings: leisure did not simply create pleasant memories; it made the city more legible. It expanded participants’ practical knowledge of Ottawa and helped them imagine a relationship to the city that extended beyond work, commuting, and obligation.

Belonging also operated within the diaspora space created by the activities themselves. The Farsi-speaking group context reduced the need for extensive self-explanation and allowed participants to feel more at ease. Shared language, shared age range, and shared migration-related concerns created culturally comfortable settings in which women could feel recognized without having to justify themselves. Even so, the data suggest that this sense of belonging remained emergent and fragile rather than fully settled, particularly when structural constraints on participation continued to operate in daily life.

### 3.6. Constraints and Facilitators

Despite the strongly positive responses to the activities, participation was consistently described as something that required active effort to make participation possible. Across the surveys, follow-up feedback sessions, and field notes, participation was largely dependent on timing, cost, transportation, weather, available energy, caregiving responsibilities, and access to clear and trusted information.

Time pressure emerged as the most persistent constraint. Participants described daily life as tightly structured around work, caregiving, household tasks, and routine obligations. One participant summarized her typical day as having ‘no gap,’ while another said simply that ‘time is short.’ A third explained that after a full work shift, ‘my body is finished.’ These comments reflect not only a shortage of time, but also the compounding effect of fatigue, scheduling pressure, and physical depletion accumulated across long days. The structured constraint checklists confirmed this pattern: lack of time was selected by 7 of 12 walk participants (58.3%), 6 of 11 dance participants (54.5%), and 7 of 9 gallery participants (77.8%).

Weather and transportation were also prominently reported as constraints. Ottawa is a four-season city, and winter conditions are demanding: daytime temperatures average roughly −6 to −12 °C and nighttime temperatures −10 to −20 °C, and the city receives an average of 240 cm of snow each year. Weather was the most frequently selected constraint in both the dance workshop (7 of 11 respondents, 63.6%) and the gallery visit (9 of 9 respondents, 100%). Transportation difficulties were particularly notable for the gallery visit, where 6 of 9 respondents (66.7%) identified them. Several participants explicitly linked attendance to winter conditions, public transit availability, and not having access to a personal vehicle. One gallery respondent wrote that she had not visited before due to ‘lack of time and not having a vehicle,’ while another noted that parking costs were a deterrent. These observations reinforce that formal availability does not guarantee practical access.

Leisure information functioned in both enabling and constraining ways. Many women learned about the activities through trusted channels such as PAND Settlement Services communications, Instagram, Telegram, and word-of-mouth within Iranian community networks. When information was clear and arrived through familiar channels, it reduced uncertainty and planning effort. When it did not, finding information became an additional layer of logistical work. One gallery respondent explained: ‘I’d rather receive emails to stay informed instead of searching on my own.’ Another said she lacked the time to follow these opportunities independently and would be more likely to attend if someone else coordinated the logistics. This highlights that the burden of locating, interpreting, and acting on leisure information is itself a meaningful access constraint. Alongside the facilitating factor of receiving leisure information through trusted channels of the settlement organization, participants indicated the no-cost fee of the activity served as a facilitator. Several women had not previously visited the National Gallery because, as one explained, “the admission fee and parking fee are too expensive,” and another noted she “didn’t know they offered free admission days.” Organizing the activity around weekly evenings with free admission—a barrier that had initially kept them away—was a facilitating factor. Having activities organized and led by someone else was also considered a facilitating factor; one woman said she did not “have time to organize anything myself, but I’d like to join activities if someone else takes the lead.” Others described the importance of not having to attend alone, with one explaining she had “never had a group of people or friends who were willing to go together” and did not enjoy such activities on her own, so a group activity made participation possible.

Taken together, the findings illustrate that participation was shaped by a continual interplay between desire and feasibility. Women valued leisure and expressed the wish for more of it, but participation depended on a constellation of practical conditions: weather, affordability, trusted communication, realistic scheduling, and logistical accessibility within already busy daily lives.

## 4. Discussion

### 4.1. Leisure, Well-Being, and Everyday Relief

For the women in this study, community-based leisure activities supported well-being in ways that are immediate, social, and grounded in the texture of everyday life. Across the three activities, leisure was valued not because it was exceptional or extravagant, but because it created temporary relief from routine, obligation, and role saturation. This aligns with research describing leisure as therapeutically valuable not merely because it is enjoyable, but because it supports restoration, coping, and positive affect [6,7,8]. It also resonates with Canadian scholarship showing that leisure contributes to immigrant women’s well-being when it creates space for enjoyment, autonomy, and social participation [8].

The present findings extend this literature by grounding the connection between leisure and well-being in the specific structure of immigrant women’s daily lives. Participants did not describe leisure in abstract health terms; they described it as time released from responsibility, time that temporarily loosened the grip of work, caregiving, repetition, and pressure. In this respect, the findings align with studies demonstrating that leisure can mediate acculturative stress and settlement distress by offering emotional replenishment, renewed meaning, and restored energy [12,13,31]. This study contributes a more grounded account of how that process was enacted through small, organized, and culturally responsive activities rather than a large-scale intervention. For research focused on mental health and wellbeing promotion among immigrant populations, this finding is promising: modest, community-embedded programming can offer immigrant women a valuable, if immediate, form of well-being support. As each activity was offered as a single session, these accounts describe immediate, self-reported responses by the women who participated in the study. Whether repeated or ongoing programming would produce more durable effects on well-being is an important question for future research.

### 4.2. Social Connection, Shared Language, and Belonging

For the women in this study, leisure also served as a social resource. Shared activities allowed women to meet others, initiate new relationships, and build familiarity through common language and common experience. This pattern is consistent with previous work showing that leisure and recreation can create low-pressure settings for social contact, inclusion, and life satisfaction among newcomers [9,20,21,32,33,34]. In this study, Farsi was not merely a communication medium; it was part of the social infrastructure of comfort. Shared language reduced the need for self-explanation and allowed participants to feel understood more readily. These findings are consistent with recent research on newcomers in a comparable Canadian urban setting that identified social connection and cultural familiarity as central motivators for leisure participation, while structural obstacles persistently constrained their ability to engage [22].

The social connection produced in these settings resonates with foundational theory on sense of community (SOC) [35]. McMillan and Chavis [35] identify four dimensions of the sense of community: membership, influence, integration and fulfillment of needs, and shared emotional connection. The experiences participants in this study described map onto all four of these dimensions The shared cultural setting, shared language, and shared life stage reduced social distance and supported a felt sense of membership. Participants described feeling that their presence mattered to the group, that the activities met social and emotional needs unaddressed elsewhere, and that the shared context of immigration created a basis for genuine connection. Recent leisure research with Korean middle-aged women found comparable dynamics, wherein culturally familiar and linguistically comfortable settings generated strong sense of community even within a single activity session [36]. Liechty and colleagues [37] similarly found that older adults in group fitness programs developed lasting sense of community through shared activity, reinforcing that brief but well-designed community leisure can initiate durable social bonds.

At the same time, our findings caution against assumptions that shared ethnicity automatically produces a sense of belonging. Some participants felt genuine warmth and recognition, while others described weaker connection or expressed a preference for more culturally mixed environments. This nuance is significant: culturally familiar programming can reduce social distance but does not override individual differences, prior social comfort, or the specific dynamics produced by any particular activity format. The results therefore support a relational view of belonging, one constructed through specific interactions and settings rather than assumed on the basis of shared identity [11,20]. An intersectional framing guides interpretation of the constraints reported in this study. Limits such as lack of time and weather are shared by many women, immigrant and non-immigrant alike. Yet for the women who participated in our study, these constraints were compounded by migration-related dimensions such as language, residency status, culture and religion, and if the participants identify as a visible minority or from a racialized community. In this sense, the experience of being an immigrant woman is not simply the sum of being a woman plus being an immigrant. The constraints these women described are best understood as shaped by intersecting dimensions of gender and migration together [2,21,24].

These leisure activities also supported place-based belonging among the study participants. Women became more familiar with Ottawa through practice: walking a route, visiting a museum, discovering free-entry programs, or entering public spaces in the company of others with shared experiences. These findings resonate with research linking immigrant leisure participation to settlement satisfaction, place familiarity, and broader social sustainability [11,12,20]. In the present study, belonging remained emergent and fragile rather than fully consolidated, particularly when structural constraints including time, transportation, and information continued to impact daily life.

### 4.3. Implications for Community-Based Wellbeing Promotion

The study’s findings carry several practical implications for mental health and wellbeing promotion programming targeting immigrant women. First, low-cost and culturally familiar leisure opportunities should be recognized as legitimate wellbeing supports rather than peripheral additions to settlement services. The consistently positive responses to the walk, dance workshop, and gallery visit, all intentionally designed with few barriers, suggest that modest and carefully designed activities can be beneficial experiences for the participants [4,11,22]. This finding adds to a growing body of evidence supporting community-based leisure and physical activity programming as a viable vehicle for health promotion among immigrant populations [10,15,38]. A related body of work further suggests that belonging is more likely to emerge when programs are designed with, rather than simply for, community members, and that co-design processes can strengthen participants’ investment in and connection to community recreation spaces [26].

Second, the constraints identified in this study reinforce research in the literature on gendered leisure constraints and immigrant participation constraints [17,18,19,20,21]. Time pressure, transportation, weather, caregiving, and information deficits are not minor inconveniences; they are the structural conditions that determine whether a potentially beneficial activity is accessible at all. Recent research on new immigrants in Montreal documented a parallel constellation of structural obstacles, underscoring that these constraints are systemic rather than individual [22], while studies of newcomers in Canadian leisure and sport programs have similarly shown that motivation to participate is insufficient when practical constraints remain unaddressed [33]. Programs aimed at supporting immigrant women’s well-being must therefore attend carefully to scheduling, seasonal considerations, location, transit access, childcare implications, and the communication burden placed on participants.

Third, trusted communication channels matter. Participants repeatedly identified PAND Settlement Services, Telegram, Instagram, and direct email as the channels through which they learned about opportunities. They also made clear that many women lacked the time and energy to search for programming independently, suggesting that community-based wellbeing promotion must not only create activities but also reduce the interpretive and logistical work required to find and act on them. Programming communicated through community-embedded networks is more likely to reach women who are already stretched by competing demands. Notably, satisfaction with the organization, communication, registration, and setting of the activities were consistently rated high across all three activities which points to features of delivery that mattered regardless of the type of activity. A consistent element was participants’ need to receive advance notice of activities taking place to plan accordingly to attend. Several women explained that they could take part more readily when information reached them early enough and through familiar channels so that they could plan attendance around work and caregiving. One participant mentioned she preferred to receive emails rather than search for activities on her own.

Finally, the variation in how participants responded to different activity formats indicates that no single leisure model will suit everyone equally well. Some women favored the informality and ease of the walk; others found deep resonance in the culturally familiar and embodied quality of dance; others valued the calm and creative atmosphere of the gallery. Programmatic diversity may therefore be a genuine asset rather than an organizational complication in leisure-based wellbeing programming for immigrant women. The growing attention to diasporic arts and cultural practices as resources for identity continuity and community building among migrants and refugees [39] further supports the case for culturally grounded and varied programming approaches.

### 4.4. Strengths and Limitations

This study brings together multiple sources of evidence, including structured surveys, open-ended written reflections, researcher field notes, and semi-structured feedback sessions, drawn from three distinct activity formats rather than a single program type. The design made it possible to examine patterns across settings and to observe how leisure experiences varied by format. The partnership with a trusted community organization enhanced accessibility and the cultural responsiveness of the study design.

Several limitations warrant acknowledgment. The sample was small and activity-based, and participants self-selected into the activities; the findings should therefore be understood as an in-depth account of a specific community-engaged project rather than as statistically generalizable results. Since participation was voluntary and shaped by the same constraints the study set out to examine, the women who took part may differ from those who were unable to attend, and the findings likely reflect the experiences of women who were able to negotiate their constraints rather than of all eligible women. As a result, the benefits reported here may understate what leisure could offer women who faced greater constraints and could not take part.

Across all three activities, some women who had initially registered did not attend. This gap between registration and attendance is itself consistent with the constraints participants described, since intending to take part did not always translate into being able to. Since the post-activity surveys were anonymous and participants could attend different combinations of activities, the survey data capture activity-level responses rather than those of a fixed cohort. Length of time in Ottawa was not systematically recorded so responses could not be analyzed by duration of residence. As most participants appeared to be recent newcomers within the last five years, the account may under-represent the experiences of women who have lived in the city longer. Beyond the sample, the study was limited in how well-being itself was captured. Well-being was measured through participants’ own accounts of how they felt during and after three specific activities, rather than through a validated well-being measure, so these findings speak to immediate, self-reported experience. Only two follow-up feedback sessions were conducted, and they were documented through notetaking rather than audio-recording. Although notes were reviewed with participants for accuracy, audio-recording might have preserved additional nuance. The study was conducted in a single Canadian city with one linguistic and cultural community, and the findings should not be assumed to apply uniformly across other immigrant women’s groups or urban contexts. The same activities offered in another season, city, or community, or with different women, could yield different responses. Future research could build on this work through larger samples, longitudinal designs, or comparative studies across multiple immigrant communities and cities.

## 5. Conclusions

For the women who took part in this study, community-based leisure activities supported well-being in ways that allowed social connection, sense of community, and familiarity with place. For the women in this study, leisure was not peripheral to settlement. It offered relief from obligation, opportunities to meet others, a growing familiarity with Ottawa, and a deepening sense that they could inhabit the city more confidently and fully. Yet these benefits were not automatic. They depended on whether activities were low-cost, clearly communicated, culturally comfortable, and practically accessible within lives shaped by work, caregiving, fatigue, weather, and transit constraints.

Leisure-based programming should be understood as a practical and relational form of wellbeing promotion that can help immigrant women feel less isolated, more socially connected, and more at ease within their communities of settlement. For practitioners, policymakers, and researchers working in immigrant mental health and wellbeing, these findings underscore the value of partnership-based, culturally responsive, and access-centered approaches. Small-scale, locally embedded activities can contribute to immigrant women’s well-being when designed with careful attention to the conditions that would enhance the possibility of participation. The leisure activities implemented in this study contributed to the well-being and sense of belonging of the immigrant women who participated, with implications for their perception of mental health and well-being promotion.

## Figures and Tables

**Table 1:** Overview of the three community-based leisure activities, including date, location, main features, and number of post-activity evaluation surveys completed.

Activity	Date	Location	Main Features	Completed Surveys
Group walk	26 October 2025	Rideau Canal/National Arts Centre	Relaxed outdoor walk followed by informal tea and conversation	12
Classical Persian dance workshop	19 November 2025	Community Center,	Culturally familiar group dance session with refreshments and informal conversation	11
National Gallery visit	4 December 2025	National Gallery of Canada	Group museum visit during Free Thursday Nights with social and creative programming	9

**Table 2:** Selected post-activity evaluation findings from structured survey items, showing positive participation ratings, social connection outcomes, and notable activity-specific results.

Activity	Positive Participation Ratings	Social Connection Findings	Notable Activity-Specific Finding
Group walk	12/12 enjoyed the activity; 12/12 found registration and participation easy; 11/12 said the walk fit their schedule	12/12 reported making at least one new acquaintance; 9/12 wanted to stay connected through a small group	6/12 reported it was their first time on the route; 11/12 said they would do the walk or a similar activity again
Dance workshop	11/11 enjoyed the activity; 11/11 were satisfied with facilitation; 10/11 felt the pace and level were suitable	11/11 reported making at least one new acquaintance	8/11 said the workshop increased their awareness of leisure opportunities in the community and city
Gallery visit	9/9 enjoyed the visit; 8/9 said the activity fit their schedule and the environment supported participation	8/9 reported enjoyable interactions with other women; 7/9 felt more connected with the Iranian community in Ottawa	8/9 said the visit increased their interest in art and culture and raised awareness of free-admission programs

**Table 3:** Frequencies and percentages of self-reported constraints on leisure participation across the three activities.

Constraint	Walk (*n* = 12)	Dance Workshop (*n* = 11)	Gallery Visit (*n* = 9)
Lack of time	7 (58.3%)	6 (54.5%)	7 (77.8%)
Family duties	6 (50.0%)	6 (54.5%)	2 (22.2%)
Childcare responsibilities	3 (25.0%)	4 (36.4%)	3 (33.3%)
Transportation	4 (33.3%)	4 (36.4%)	6 (66.7%)
Weather	3 (25.0%)	7 (63.6%)	9 (100.0%)
Not knowing local leisure resources	8 (66.7%)	3 (27.3%)	3 (33.3%)
Cost	2 (16.7%)	3 (27.3%)	2 (22.2%)

## Data Availability

The data presented in this study are not publicly available due to the qualitative nature of the material and the requirement to protect participant confidentiality given the small, community-based sample. Data are available on request from O’Connor as per university safe data storage policies.
